# Spatial progression and molecular heterogeneity of IDH-mutant glioblastoma determined by DNA methylation-based mapping

**DOI:** 10.1186/s40478-021-01221-7

**Published:** 2021-06-30

**Authors:** James F. Lyon, Varshini Vasudevaraja, Kanish Mirchia, Jamie M. Walker, Robert J. Corona, Lawrence S. Chin, Ivy Tran, Matija Snuderl, Timothy E. Richardson, Mariano S. Viapiano

**Affiliations:** 1 Department of Neurosurgery, State University of New York, Upstate Medical University, 750 E. Adams St, IHP 4604, Syracuse, NY 13210 USA; 2 Department of Pathology, New York University Langone Health, New York City, NY 10016 USA; 3 Department of Pathology, State University of New York, Upstate Medical University, Syracuse, NY 13210 USA; 4 Department of Pathology and Laboratory Medicine, University of Texas Health San Antonio, 7703 Floyd Curl Dr., MC 8070, San Antonio, TX 78229 USA; 5 Glenn Biggs Institute for Alzheimer’s and Neurodegenerative Diseases, University of Texas Health San Antonio, 7703 Floyd Curl Dr., MC 8070, San Antonio, TX 78229 USA; 6 Department of Neuroscience and Physiology, State University of New York, Upstate Medical University, 750 E. Adams St, IHP 4604, Syracuse, NY 13210 USA

**Keywords:** Astrocytoma, Glioblastoma, DNA methylation profiling, Copy number profiling, IDH-mutation, *MGMT* methylation

## Abstract

Glioblastoma (GBM) is the most common malignant primary central nervous system (CNS) neoplasm in adults, and has an almost universally poor prognosis. Recently, an emphasis on genetic and epigenetic profiling has revealed a number of molecular features useful in the diagnostic and prognostic classification of GBM, advancing our understanding of the underlying features that make these tumors so aggressive and providing the rationale for the creation of better targeted therapeutics. One such method, DNA methylation profiling, has recently emerged as an important technique for the classification of CNS tumors, with diagnostic accuracy in some cases surpassing traditional methods. However, how DNA methylation profiles change with the course of the disease remains less understood. Here, we present a case of a 30-year-old male with primary IDH-mutant GBM with widespread recurrence and death two years later. Using unsupervised hierarchical clustering of methylation probes, we created a phylogenetic map to trace the tumor path as it spread from the initial biopsy site throughout the right hemisphere, across the corpus callosum to the contralateral hemisphere, and into the brainstem. We identified molecular divergence between the right and left hemisphere GBM samples marked by distinct copy number profile alterations, alterations in specific methylation sites, and regional loss of *MGMT* promoter methylation, providing a potential mechanism for treatment resistance in this case. In summary, this case both highlights the molecular diversity in GBM, and illustrates a novel use for methylation profiling in establishing a phylogenetic profile to allow for spatial mapping of tumor progression.

**Supplementary Information:**

The online version contains supplementary material available at 10.1186/s40478-021-01221-7.

## Introduction

Glioblastoma (GBM) is the most common and most aggressive form of glioma and carries an almost universally poor prognosis [20, 28]. Historically, these tumors have been classified and graded based solely on their histologic morphology, as densely cellular, pleomorphic tumors with mitotic activity and either microvascular proliferation and/or necrosis, the latter two features being powerful predictors of aggressive tumor behavior [19]. These histologic features are indicative of high tumor cell proliferation and metabolic demand, which requires tumor cells to modify their micro-environment or find a route of escape to survive. The interplay between hypoxia, necrosis, growth factor expression, tumor micro-environment, and malignant clone selection provides a possible explanation for the marked molecular heterogeneity observed in glioblastomas [2–4, 36]. As of the 2016 revised 4th edition of the *WHO Classification of Tumours of the Central Nervous System*, classification of diffuse gliomas involves consideration of both histopathologic features as well as molecular and genetic features, including 1p/19q codeletion status and mutations in isocitrate dehydrogenase 1 and 2 (*IDH1/2*). *IDH1/2* mutations define the subclasses of IDH-wildtype and IDH-mutant GBM, the latter of which occurs in significantly younger patients and generally carries a more favorable prognosis [20]. Since the publication of the 2016 WHO guidelines, numerous studies have investigated additional clinical, radiologic, and molecular prognostic factors in an effort to further subclassify both IDH-wildtype and IDH-mutant gliomas and improve diagnostic and prognostic categories [26].

Genome-wide DNA methylation profiling has proven to be a particularly robust and reproducible tool in central nervous system (CNS) tumor classification and has been increasingly utilized as a marker of cell development and a surrogate representation of gene expression, which is particularly helpful in cases where the histology is unusual or non-specific and/or other molecular testing is inconclusive [16, 30]. DNA methylation profiling measures epigenetic alterations in which methyl groups are transferred to the 5′ position of the cytosine ring, forming CpG islands throughout the genome in patterns that can be measured to classify CNS tumors [5, 41]. Additionally, DNA methylation can inactivate tumor suppressor genes and/or cause genomic instability and push cells toward malignant progression, and may also partially explain the molecular heterogeneity found in GBM [10, 18, 23, 29, 37]. One gene for which methylation status is of particular interest is *O6-methylguanine-DNA methyl-transferase* (*MGMT*), which is a predictive biomarker of tumor response to the standard-of-care chemotherapeutic agent for GBM, temozolomide [8, 24]. While the value of DNA methylation for diagnosis is well-established, how DNA methylation changes within the same tumor over the progression of the disease remains to be explored.

In this report, we analyzed the original biopsy and subsequent numerous spatially diverse autopsy samples in a case of IDH-mutant GBM with *MGMT* methylation in a 30-year-old patient who experienced relatively rapid recurrence and short survival interval, using genome-wide methylation profiling and copy number profiling in addition to standard histopathological techniques. In addition, we leveraged differences in epigenetic alterations in the methylation profiles at the individual probe level to produce unsupervised hierarchical clustering, determining the “molecular distance” among individual samples and reconstructing the hypothetical spatial path taken by the tumor as it invaded the brain.

## Methods

### Tissue sampling

The primary tumor resection specimen and recurrent tumor post-mortem specimens (obtained within 2 h of death) were processed according to standard protocols. At autopsy, the right hemisphere was sectioned in the coronal plane to better evaluate the resection cavity and the left hemisphere was sectioned in the sagittal plane to evaluate the tumor spread. Extensive sampling was performed in the bilateral cortex, including sections from the original resection specimen in right temporal lobe (sample 1), tumor resection cavity wall in right temporal lobe (sample 2), right parietal lobe (sample 3), right occipital lobe (sample 4), right frontal lobe/corpus callosum (sample 5), left frontal lobe/corpus callosum (sample 6), left superior frontal lobe (sample 7), left lateral frontal lobe (sample 8), and midbrain (sample 9).

### Histology and immunohistochemistry

H&E-stained slides were prepared from 4 μm thick sections of formalin-fixed, paraffin-embedded (FFPE) tissue using standard protocols. Immunohistochemistry was performed on 4 μm paraffin sections following heat-induced epitope retrieval using CC1 (Ventana, Tucson, AZ, USA), then staining with GFAP (Thermo Fisher Scientific, Waltham, MA, USA), IDH1 R132H (Dianova, Hamburg, Germany), ATRX (Sigma-Aldrich, St. Louis, MO, USA), p53 (Ventana), and Ki-67 (Dako, Carpinteria, CA, USA) on either a Ventana Benchmark XT or Ventana Benchmark Ultra automated stainer, using Ventana UltraView Universal DAB Detection kits (Ventana).

### DNA methylation analysis and deconvolution

DNA extraction from 10 FFPE slides on each of the 9 samples was carried out using the automated Maxwell system (Promega, Madison, WI, USA). DNA methylation was analyzed by the Illumina EPIC Human Methylation array, assessing 850,000 CpG sites (Illumina, San Diego, CA, USA), according to the manufacturer’s instructions at the NYU Molecular Pathology laboratory, as previously described [38]. Molecular subtype classification and t-Distributed Stochastic Neighbor Embedding (t-SNE) visualization was performed utilizing the cloud-based DNA methylation classifier, and tumors were classified with the methylation classifier previously developed for CNS tumors (www.molecularneuropathology.org) [5]. In addition, the array data were used to calculate a low-resolution copy number profile (CNP), with gains and losses noted relevant to baseline, also previously described [12, 15, 27, 32, 34, 35, 39, 42, 43]. “Amplification” in the copy number profile was determined by log2 ≥ 0.3. The data were analyzed using the R package (http://www.R-project.org/) in Bioconductor. Unsupervised hierarchical clustering of the top 10,000 differently expressed DNA methylation regions was performed on each sample [41]. Differential methylation analysis between each pair of clusters (C1: sample 1; C2: samples 2, 3, 4, 9; C3: samples 5, 6, 7, 8) was performed by pairwise comparison in order to identify differentially methylated sequences with FDR < 0.01. Probes were filtered using previously described methods to identify regions corresponding to gene bodies, untranslated regions, promoters, and upstream regulatory regions (up to 1.5 kb upstream of each gene) [1]. For each pairwise comparison between clusters, the top 50 hypermethylated and hypomethylated genes were identified using the R package ComplexHeatmap. Venn diagrams were used to visualize common and unique genes between clusters, both in hypermethylated and hypomethylated categories. Gene set enrichment analysis (GSEA) was performed using the probes matching gene body and promoter regions for each methylation cluster, to identify differentially activated pathways between clusters (https://www.gsea-msigdb.org/gsea/index.jsp) [40]. MethyCIBERSORT was used to deconvolve the cell populations in the microenvironment of each cluster, including endothelial, fibroblast, and immune cell populations, as previously described [6, 41].

### Brain atlas mapping

Reference MRI brain images encompassing the coordinates of the original tumor location were downloaded from the Allen Brain Atlas MRI project https://portal.brain-map.org/ (Allen Institute, Seattle, WA, USA; the image set H0351.1012 was chosen for being age-matched to the patient). MRI images were processed using MRIcron software and annotated by hand using detailed photographs from the brain autopsy tumor sampling.

## Results

### Case history

**Fig. 1:**
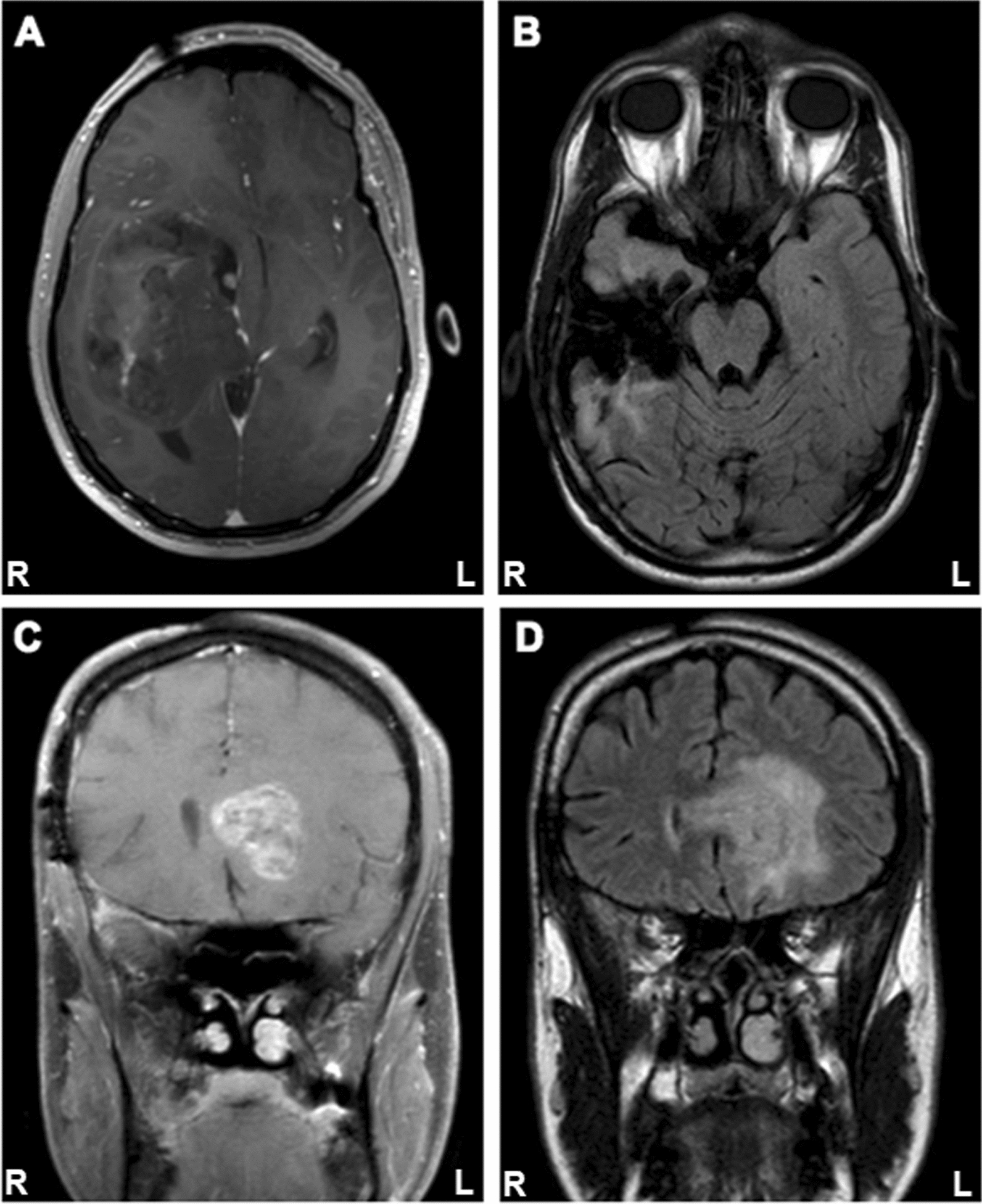
MRI panels demonstrating **A** gadolinium contrast enhancing T1 of initial tumor centered in right temporal/parietal lobe with right-to-left shift (transverse section), **B** the resection cavity immediately following surgery (transverse section), **C** gadolinium contrast enhancing T1 of recurrent tumor with migration to left hemisphere (coronal section), and **D** T2 FLAIR imaging of recurrent tumor with migration to left hemisphere (coronal section)

A 30-year-old male patient presented to the emergency room with complaints of worsening headache, nausea, and increased somnolence, where he was discovered to have left-sided hemiparesis, including facial droop and arm drift. Subsequent imaging studies demonstrated a large, focally enhancing right frontotemporal lobe mass with extensive mass effect, 1.1 cm midline shift, and marked right uncal herniation (Fig. 1A). The patient was taken for craniotomy and resection of the frontotemporal mass. Brain imaging following the procedure showed postoperative change consistent with tumor debulking and near complete tumor resection (Fig. 1B). The tumor consisted of a densely cellular astrocytic neoplasm with gemistocytic features (Additional file 1: Figure S1A), scattered mitotic figures, necrosis, and microvascular proliferation (Additional file 1: Figure S1B), consistent with glioblastoma, WHO grade IV. The tumor cells were positive for GFAP and IDH1 R132H (Additional file 1: Figure S1B, C). There was loss of ATRX, increased nuclear p53 staining, and significantly elevated Ki-67 proliferation index (Additional file 1: Figure S1D–F). The tumor was negative for 1p/19q codeletion. Repeat imaging one month after surgery demonstrated new enhancing lesions at the margins of the surgical cavity and in the perivascular spaces; accordingly, the patient was prescribed a course of temozolomide and localized brain irradiation.

Eighteen months later, the patient returned with complaints of left-sided weakness, numbness, and severe right-sided headache. Repeat imaging demonstrated widespread tumor growth with multifocal enhancing masses adjacent to the original resection cavity, right parietal and occipital lobe, and extending across the corpus callosum into the left frontal lobe (Fig. 1C, D). Surgical intervention was declined by the patient, who repeated the temozolomide and irradiation treatment, adding bevacizumab and olaparib adjuvant therapy. Ultimately the patient passed away in the hospice setting two years after the initial diagnosis.

**Fig. 2:**
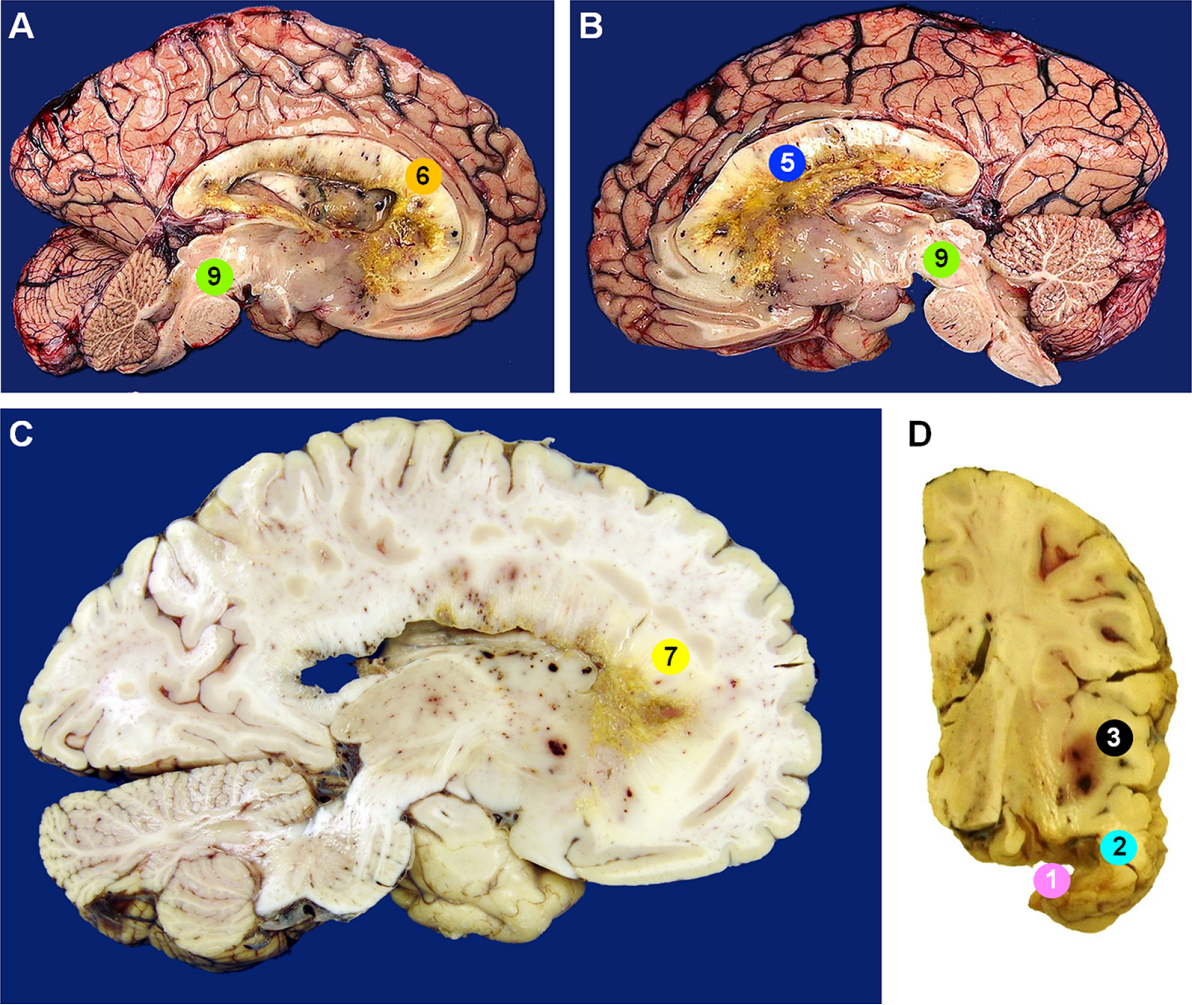
Gross images of autopsy specimens demonstrate significant tumor growth extending throughout the corpus callosum and brainstem in the **A** left hemisphere and **B** right hemisphere, as well as in the **C** left frontal lobe, and **D** right temporal and parietal lobes

**Fig. 3:**
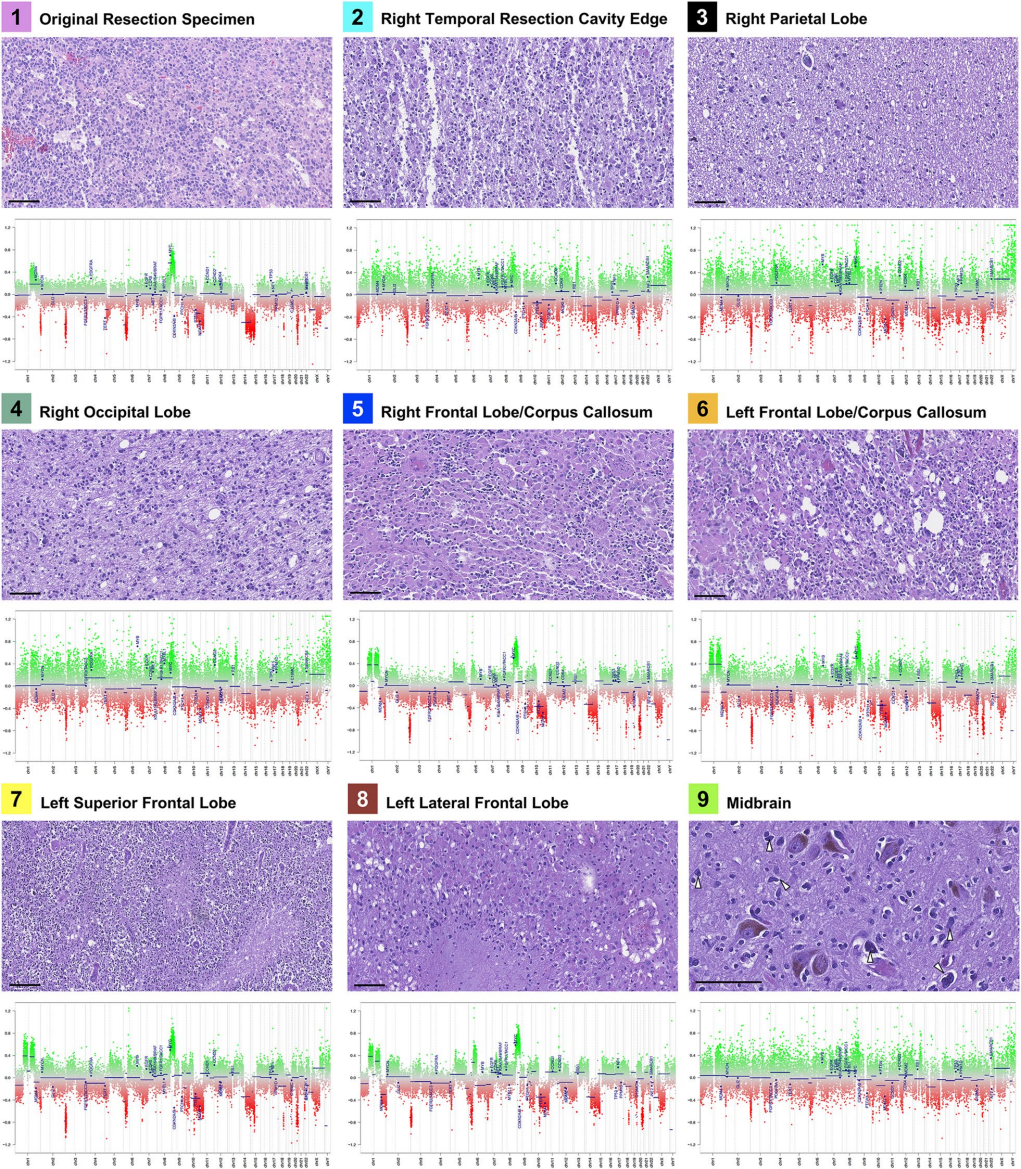
H&E images of each of the original resection specimen as well as the 8 profiled regions of tumor taken at autopsy along with their corresponding copy number profiles. All histologic images are taken at a total magnification of 200x, scale bars = 100 μm

At brain-only autopsy, there was extensive infiltration throughout the bilateral cerebral hemispheres with necrotic tumor grossly identified in the tumor resection cavity, right frontal, parietal, temporal, and occipital neocortex, along the bilateral lateral ventricles, throughout the corpus callosum, left frontal neocortex, and brainstem (Fig. 2). Histologic examination of each region (Fig. 3) demonstrated a variably fibrillary to gemistocytic astrocytic tumor with infiltration around neurons, frequent mitotic figures, microvascular proliferation, and necrosis. Immunohistochemical analysis of multiple autopsy regions revealed an identical profile to the initial resection specimen.

### Global methylation analysis and t-SNE

**Fig. 4:**
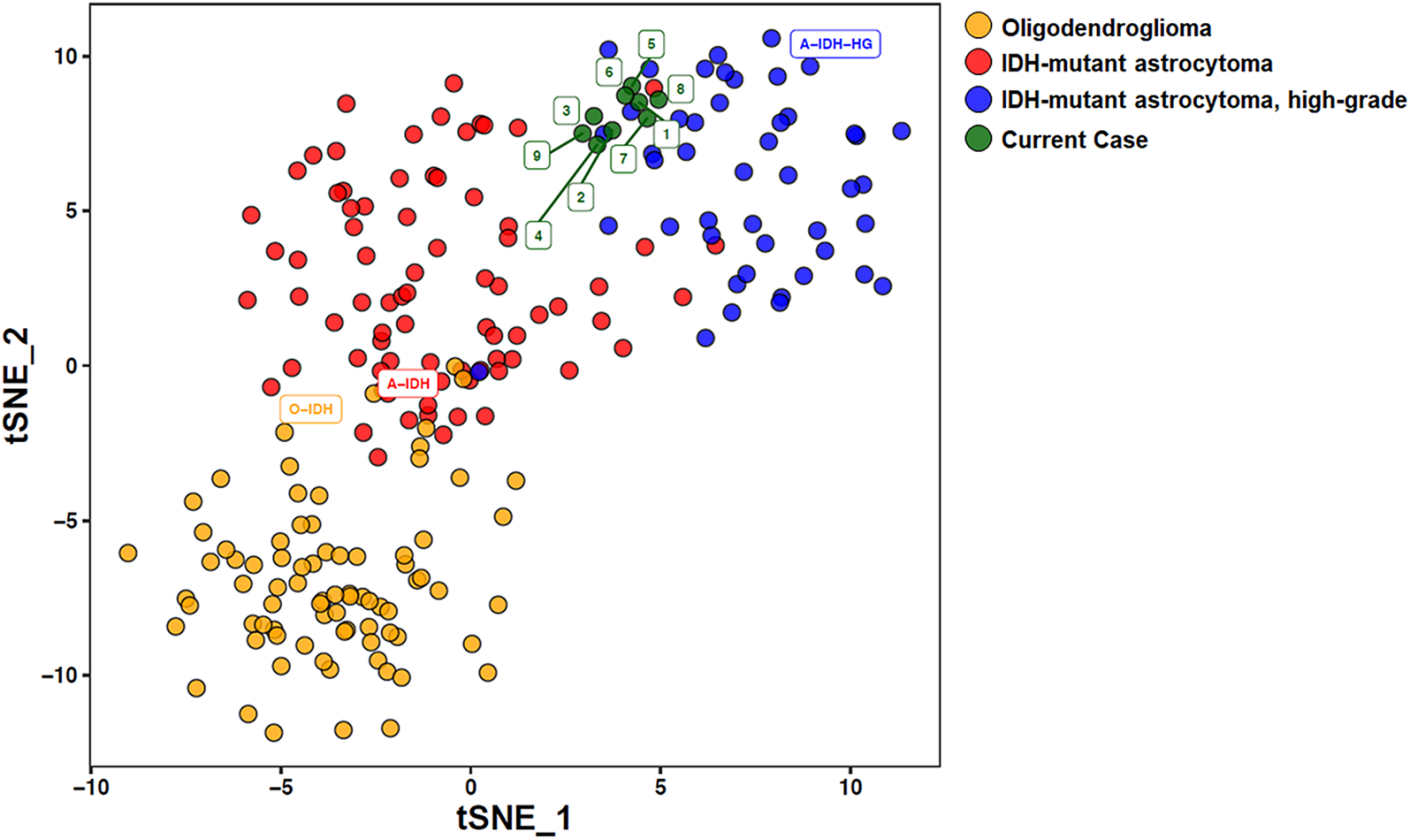
t-Distributed Stochastic Neighbor Embedding (t-SNE) plotting demonstrating the location of the initial biopsy (specimen 1) in relation to methylation profiles of the autopsy samples (specimens 2–9) and oligodendroglioma, IDH-mutant astrocytoma, and high-grade IDH-mutant astrocytoma subsets.

Global methylation profiling (https://www.molecularneuropathology.org/mnp) [5] classified the initial resection specimen as IDH-mutant astrocytoma, high-grade (class score 0.9834). Profiling of 8 additional tumor regions demonstrated very similar results for each of the samples profiled, indicating with high confidence that all samples were from the same neoplastic process (Fig. 4). There were two apparent subclusters within the high-grade IDH-mutant astrocytoma grouping, one containing the original resection (sample 1) and recurrent tumor masses from the corpus callosum and left hemisphere (samples 5, 6, 7, and 8), and a second group containing the recurrent tumor masses adjacent to the original resection in the right hemisphere, and the midbrain (samples 2, 3, 4, and 9).

### Copy number profiling

Total copy number profiling demonstrated three distinct groups of copy number variation (CNV) in the tumor specimens. The first group, composed of the original tumor resection, showed relatively mild generalized CNV with focal gains in 1q and 8q, and focal losses at 2p, 3p, 5p, 6p, 9p, 10, 11, 14q, 15, and 20q (Fig. 3). Notably, this includes loss of *CDKN2A* and *PTEN*, and gain of *MDM4*, *MYC*, and *PDGFRA*. A second group was formed by samples 2–4 in the right hemisphere, closely associated with the original resection, and to a lesser extent sample 9 from the midbrain. A third group, comprised of the recurrent masses in the contralateral hemisphere, was defined by more discrete alterations, including twin gains in 1p and 1q, gain in 8q, and losses in 3p, 6p, 10q, 14, and 20q (Fig. 3).

### Methylation profiling clustering and brain mapping

**Fig. 5:**
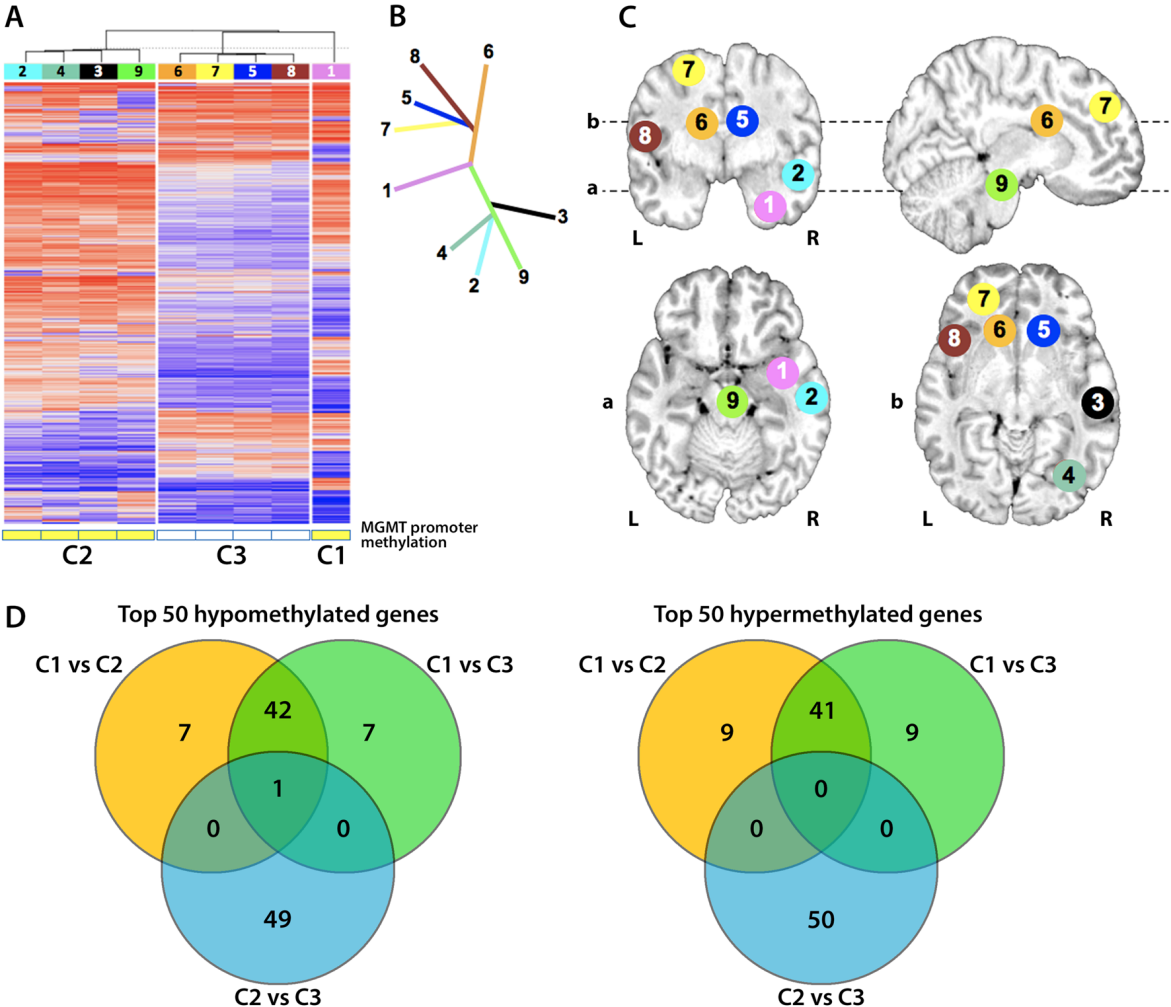
Clustering of samples 1–9 based on their top 10,000 most differently methylated probes. **A** Unsupervised hierarchical clustering revealed 3 distinct clusters: the original tumor resection in the right temporal lobe (C1: sample 1), tumor in the ipsilateral cerebral hemisphere and brainstem (C2: samples 2, 3, 4, and 9), and tumor in the corpus callosum and contralateral cerebral hemisphere (C3: samples 5–8). **B** Phylogenetic relationship of each sample. **C** Spatial location of each tumor sample on standard MRI overlays. **D** Top-50 hyper- and hypo-methylated genes determined by pairwise comparison between clusters. Comparisons (C1 vs. C2) and (C1 vs. C3) share most of the hyper- and hypomethylated genes, whereas the comparison (C2 vs. C3) yields a separate set of differentially methylated genes

To further classify these tumors and establish a more robust molecular spatial profile, we analyzed the top 10,000 most different methylation probes across the entire genome [41], and performed unsupervised hierarchical clustering (Fig. 5A). This method recapitulated the same three groups identified by CNV: the original tumor (cluster 1: sample 1) diverged at recurrence into two separate groups, one containing the samples around the original resection specimen and extending into the midbrain (cluster 2: samples 2–4 and sample 9), and a separate group in the right corpus callosum, left frontal lobe/corpus callosum, and left frontal lobe (cluster 3: samples 5–8). We did not observe significant differences in the distribution of methylation probes corresponding to gene bodies, promoters, or regulatory regions between clusters (Additional file 1: Figure S2). Hierarchical clustering based around these 10,000 individual methylation probes allowed us to construct a hypothetical spatial and directional assessment of tumor spread, matching anatomical locations (Fig. 5B, C).

A notable feature revealed by methylation analysis was the status of *MGMT* promoter methylation (Fig. 5A). The original tumor (cluster 1, C1), as well as recurrent masses directly adjacent to the tumor resection cavity and in the midbrain (cluster 2, C2) showed *MGMT* promoter methylation, whereas samples anatomically distant from the original tumor and outside of any radiation field (cluster 3, C3) lost this *MGMT* promoter methylation. This provides a potential explanation for the aggressive behavior of the recurrent tumor, in that loss of *MGMT* promoter methylation could have favored resistance to temozolomide and other alkylating agents. This difference in *MGMT* methylation profiles also suggests two separate evolutionary pathways in the tumor, corresponding with the differences in CNV profiling and the methylation hierarchical clustering.

### Gene set enrichment analysis (GSEA) and tumor microenvironment deconvolution

Genes showing the highest differences in methylation between each pair of clusters (by pairwise comparison) were visualized using ComplexHeatmap (Additional file 1: Figure S3 and Table S1). A majority of these genes were consistently hyper- or hypomethylated in the original tumor compared against each of the recurrent clusters (comparisons C1 vs. C2 and C1 vs. C3, Fig. 5D). However, the comparison between the two recurrent clusters (C2 vs. C3) revealed entirely different sets of hyper- and hypo-methylated genes, in agreement with the lower similarity between these two clusters compared with their respective similarity to C1. Despite the presence of differentially methylated genes between the primary tumor and the recurrent clusters, we were unable to identify significantly different (FDR < 0.01) signaling pathways when comparing C1 versus C2 or C1 versus C3. We therefore focused on analyzing the differences between C2 and C3, which presented larger phylogenetic divergence to each other than to the original tumor. Using GSEA we identified differences between these clusters regarding cell cycle; oncogenic drivers (“bladder cancer” signature); complement activation and presence of immune cells (“hematopoietic cell lineage”); cell autophagy (“mTOR signaling” and “mitophagy” signatures); and mRNA surveillance (Additional file 1: Figure S4). Of these, mTOR signaling and mRNA surveillance pathways were not significantly different after correcting for multiple comparisons.

Further analysis of differential methylation between clusters using MethylCIBERSORT allowed us to explore the tumor microenvironment by deconvolving the signatures of different types of tumor-associated cells [6, 41]. We found measurable differences in immune epigenetic signatures between the three clusters (Additional file 1: Figure S5). C2 (proximal recurrence) demonstrated significantly increased presence of CD4 effector cells (*p* = 0.041) and neutrophils (*p* = 0.046) compared to the other two clusters, as well as significantly decreased epigenetic signatures for endothelial cells (*p* = 0.036) and CD19 (*p* = 0.035), CD8 (*p* = 0.035), and regulatory T-lymphocytes (*p* = 0.026). C3 (distal recurrence) demonstrated a significantly increased epigenetic signature for fibroblasts compared to clusters 1 and 2 (*p* = 0.046).

## Discussion

Despite extensive advances in surgical management and novel molecular-targeted therapies of GBM, these aggressive tumors continue to have an almost uniformly dismal prognosis [9, 11]. While cases of GBM with mutations in *IDH1* and *IDH2* have significantly better clinical outcomes in terms of progression-free survival and overall survival than their IDH-wildtype counterparts, the vast majority of these patients still die from their disease within 5 years, and “long-term survival” is often defined as survival ≥ 36 months [31]. Since a hallmark of GBM cells is their infiltration and diffuse spread through native CNS tissue, these tumors are usually considered to be surgically incurable; furthermore the significant intratumoral molecular heterogeneity of GBM may result in the evolution of aggressive and therapy-resistant clones, which defeat adjuvant treatments [10, 25, 29, 33, 37, 45].

In this report, we evaluated the original biopsy and numerous spatially diverse autopsy samples in a case of IDH-mutant GBM with *MGMT* methylation in a 30-year-old male patient with relatively rapid recurrence and short survival. Each sample was analyzed using genome-wide methylation profiling and copy number profiling in addition to standard histopathological techniques. All samples grouped together as IDH-mutant astrocytoma, high-grade, by the brain tumor classifier and t-SNE plotting (Fig. 4); however, more detailed analysis revealed measurable molecular divergence and subclustering between the initial biopsy and the GBM masses found in the right and left hemispheres regarding their distinct copy number profile alterations, alterations in specific methylation sites, and loss of *MGMT* promoter methylation (Figs. 3, 4, 5).

This is, to our knowledge, the first report to utilize methylation profiling to create a spatial map of GBM progression through the brain, and serves as a proof-of-concept study to demonstrate the feasibility of leveraging hierarchical clustering of relatively minor differences in thousands of individual methylation probes to establish a molecular phylogeny of tumor samples and trace tumor infiltration. These methods allowed us to create a phylogenetic map tracing the tumor’s path from the initial biopsy site in the right hemisphere to the contralateral hemisphere and into the brainstem.

Our results clearly separate the tumor samples into three separate molecular clusters (Fig. 5), matching their disparate anatomic sites. These clusters, corresponding to the initial tumor (C1) and the recurrent masses adjacent (C2) and distal (C3) to the original malignance, showed significant differences in copy number profiles, *MGMT* methylation status, differentially active (hypomethylated) signaling pathways, and composition of their immune microenvironment. Phylogenetic distance between clusters suggests that the recurrent masses originated as separate events from the original tumor and progressed in different directions, resulting in higher similarity of C2 and C3 to the original tumor than each other. Pairwise comparison and pathway analysis failed to show significantly different signaling mechanisms between the original tumor and the recurrent clusters, but revealed notable differences between the two recurrent regions. For example, the cluster C2, which was MGMT-methylated and recurred in an irradiated region, had increased methylation of inflammation-related genes (Additional file 1: Figures S3, S4) and increased epigenetic signature of neutrophils (Additional file 1: Figure S5). These changes are interesting from a prognostic perspective because they could have been associated to local immunosuppression and increased tumor dispersion [17], suggesting that, despite their locoregional treatment, those tumor masses were still in the process of further progression. 

From a diagnostic standpoint, we found of particular interest the differences in *MGMT* methylation status between the original tumor and the recurrent tumor clusters. Autopsy samples that retained *MGMT* promoter methylation were spatially adjacent to the original tumor location, extending as far as the midbrain, whereas the *MGMT*-unmethylated samples were those in the contralateral hemisphere, away from the area of the brain that was irradiated. This suggests that irradiation could have allowed for the evolution of *MGMT*-unmethylated tumor cells, which “escaped” and migrated to the other hemisphere, or perhaps that those cells had already infiltrated to the corpus callosum and out of the radiation field prior to the initial irradiation, providing an explanation for the development of temozolomide resistance seen at recurrence. It is also possible that the temozolomide treatment itself promoted extensive mutation, as recently demonstrated in IDH-mutant low-grade gliomas [44], promoting the escape of resistant clones toward the contralateral hemisphere. More importantly, our data also suggest that changing molecular features (including *MGMT* promoter methylation and other key markers of aggressiveness and treatment responsiveness in diffuse gliomas) may be missed in the routine analysis of single biopsy or resection samples and that whole-genome methylation profiling and copy number profiling in multiple samples may provide useful information in the case of tumor recurrence to identify potentially useful changing molecular milieu. It is also worth noting that the original tumor had homozygous loss of *CDKN2A,* which has prognostic relevance in patients with histologically confirmed GBM in addition to lower-grade astrocytomas. This loss could have overcome the beneficial prognostic effect of the IDH1 mutation and may in part explain the initial aggressive growth, early recurrence, and short overall survival in this patient [22, 31].

In conclusion, these data give insight into the mechanisms that can lead to resistance to tumor recurrence and resistance to therapy, highlight the molecular diversity in GBM with progression, and illustrate a novel use for methylation profiling in establishing a phylogenetic profile to allow for spatial mapping of tumor progression. As molecular profiling becomes the standard of care in the diagnosis and treatment of CNS neoplasms and personalized therapies become the norm [13, 20, 21, 26], molecular heterogeneity between tumor cells or tumor regions is increasingly important when attempting to predict clinical outcome and response to treatment. Recent studies have shown that important molecular alterations can potentially be identified relatively inexpensively and in approximately the time it takes to perform standard histologic and immunohistochemical workups [7, 14], so it is conceivable that in the near future, molecular analysis of multiple regions of infiltrating gliomas may provide a more complex and nuanced picture of the underlying biology and evolution of these tumors, and may aid in more accurate prediction of therapeutic response.

## Supplementary Information

**Additional file 1:** Supplementary materials containing figures S1 to S5 and table S1.

## Data Availability

the methylation profiling data presented in this paper will be made freely available upon reasonable request.
