# Supplementary material for: Spatial progression and molecular heterogeneity of IDH-mutant glioblastoma determined by DNA methylation-based mapping
Source: Acta Neuropathol Commun. 2021 Jun 30;9:120. doi: 10.1186/s40478-021-01221-7 (PMC8243907; doi:10.1186/s40478-021-01221-7)
Supplement: Supplementary file 1 — Additional file 1: Supplementary materials containing figures S1 to S5 and table S1. [file 40478_2021_1221_MOESM1_ESM.pdf]

Additional File - Supplementary Information

Spatial Progression and Molecular Heterogeneity of IDH-Mutant Glioblastoma Determined by DNA Methylation-Based Mapping

James F. Lyon, BS[1], Varshini Vasudevaraja, MS[2], Kanish Mirchia, MD[3], Jamie M. Walker, MD, PhD[4,5], Robert J. Corona, DO[3], Lawrence S. Chin, MD[1], Ivy Tran, BS[2], Matija Snuderl, MD[2], Timothy E. Richardson, DO, PhD[3,4,5,7,8], Mariano S. Viapiano, PhD[1,6,7,8]

[1] Department of Neurosurgery, State University of New York, Upstate Medical University, Syracuse (NY 13210); [2]Department of Pathology, New York University Langone Health, New York City (NY 10016); [3] Department of Pathology, State University of New York, Upstate Medical University, Syracuse (NY 13210); [4] Department of Pathology and Laboratory Medicine, University of Texas Health San Antonio, San Antonio (TX 78229); [5] Glenn Biggs Institute for Alzheimer's & Neurodegenerative Diseases, University of Texas Health San Antonio, San Antonio (TX 78229); [6] Department of Neuroscience and Physiology, State University of New York, Upstate Medical University, Syracuse (NY 13210)

[7] These authors contributed equally.

[8] Corresponding authors:

Mariano S. Viapiano, PhD
Department of Neurosurgery
Department of Neuroscience and Physiology
State University of New York, Upstate Medical University
750 E. Adams St, IHP 4604, Syracuse, NY, USA 13210
viapianm@upstate.edu

Timothy E. Richardson, DO, PhD
Department of Pathology and Laboratory Medicine
Glenn Biggs Institute for Alzheimer's & Neurodegenerative Diseases
UT Health San Antonio
7703 Floyd Curl Dr., MC 8070, San Antonio, TX, USA 78229
richardsont2@uthscsa.edu

Content: Supplementary Figures S1 to S5

Supplementary Table S1

Supplemental Figure S1. Histology of the initial tumor resection. **(A)** H&E demonstrates a significantly hypercellular infiltrating neoplasm, **(B)** GFAP staining demonstrates strong diffuse staining in tumor cells and highlights areas of microvascular proliferation in negative relief (arrowheads), **(C)** IDH1 R132H stain demonstrates strong, diffuse staining, **(D)** ATRX staining is negative in tumor nuclei, suggesting *ATRX* mutation, **(E)** p53 is significantly increased in tumor cell nuclei, suggesting *TP53* mutation, **(F)** Ki-67 proliferation index is significantly elevated. All histologic images are taken at a total magnification of 200x, scale bars = 100μm.

Supplemental Figure S2. Probes showing differential methylation between clusters (as determined by pairwise comparisons with FDR < 0.1) were filtered using TSS200 and TSS1500 in order to assign those probes to promoters, gene bodies, untranslated regions, and regions up to 200 and 1500 bp upstream of the transcriptional start (AvgBeta>0.8 was taken as hypermethylated and AvgBeta<0.2 as hypomethylated). No differences were seen in the genomic distribution of probes for each cluster-to-cluster comparison.

Supplemental Figure S3. Heatmap demonstrating the 50 most hypermethylated and 50 most hypomethylated genes between each of the three clusters, determined by pairwise comparison. Most hyper- and hypo-methylated genes were shared in the comparisons (C1 vs C2) and (C1 vs C3) but were entirely different from those found in the comparison (C2 vs C3).

Supplemental Figure 4. Gene set enrichment analysis (GSEA) identified significant differences between sample clusters C2 and C3 (after correcting for multiple comparisons) in gene signatures for cell cycle, oncogenic drivers ("bladder cancer" signature), complement and coagulation cascades, hematopoietic cell lineage, spliceosome, and mitophagy. Gene signatures for mTOR

signaling and mRNA surveillance pathways were not significantly different after correcting for multiple comparisons.

Supplemental Figure 5. Box plots showing methylCIBERSORT-based deconvolution of methylation profiles to identify cell types in the microenvironment of clusters C1, C2, and C3. Relative abundance of epigenetic signatures for monocyte lineage (*CD14*), B-lymphocytes (*CD19*), CD4 effector T-lymphocytes (*CD4_Eff*), NK cells (*CD56*), cytotoxic T lymphocytes (*CD8*), endothelial cells, eosinophils (*Eos*), fibroblasts, neutrophils (*Neu*), and regulatory T cells (*Treg*) are illustrated on each plot. Kruskal-Wallis test was used to identify significant differences between clusters.

Supplemental Table I: 50 most hypermethylated and 50 most hypomethylated genes determined by pairwise comparison between clusters (as shown in Figure 5D and Supplemental Figure S3). The table shows the genes that are unique to a given comparison (C1 vs C2; C1 vs C3; C2 vs C3) or common between these comparisons.

Figure S1

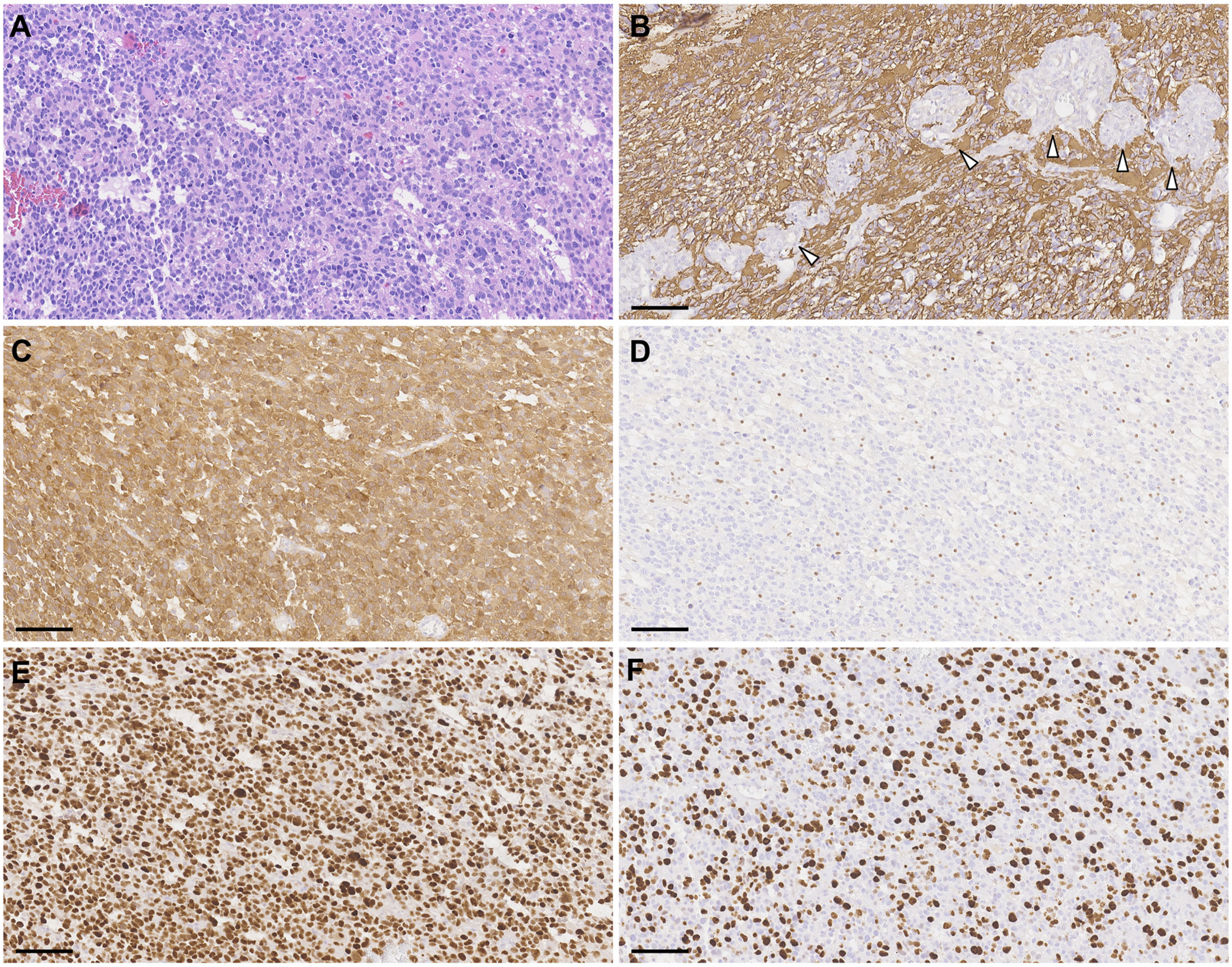

Figure S2

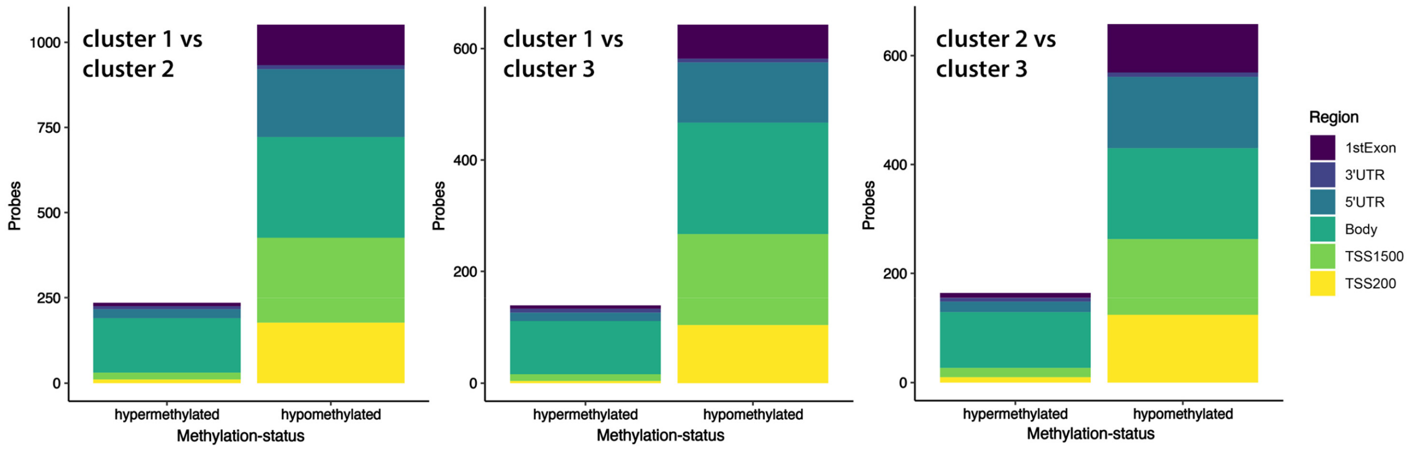
cluster 1 vs
cluster 2
cluster 1 vs
cluster 3
cluster 2 vs
cluster 3
Probes
0
250
500
750
1000
0
200
400
600
hypermethylated
hypomethylated
Methylation-status
Region
1stExon
3'UTR
5'UTR
Body
TSS1500
TSS200

Figure S3

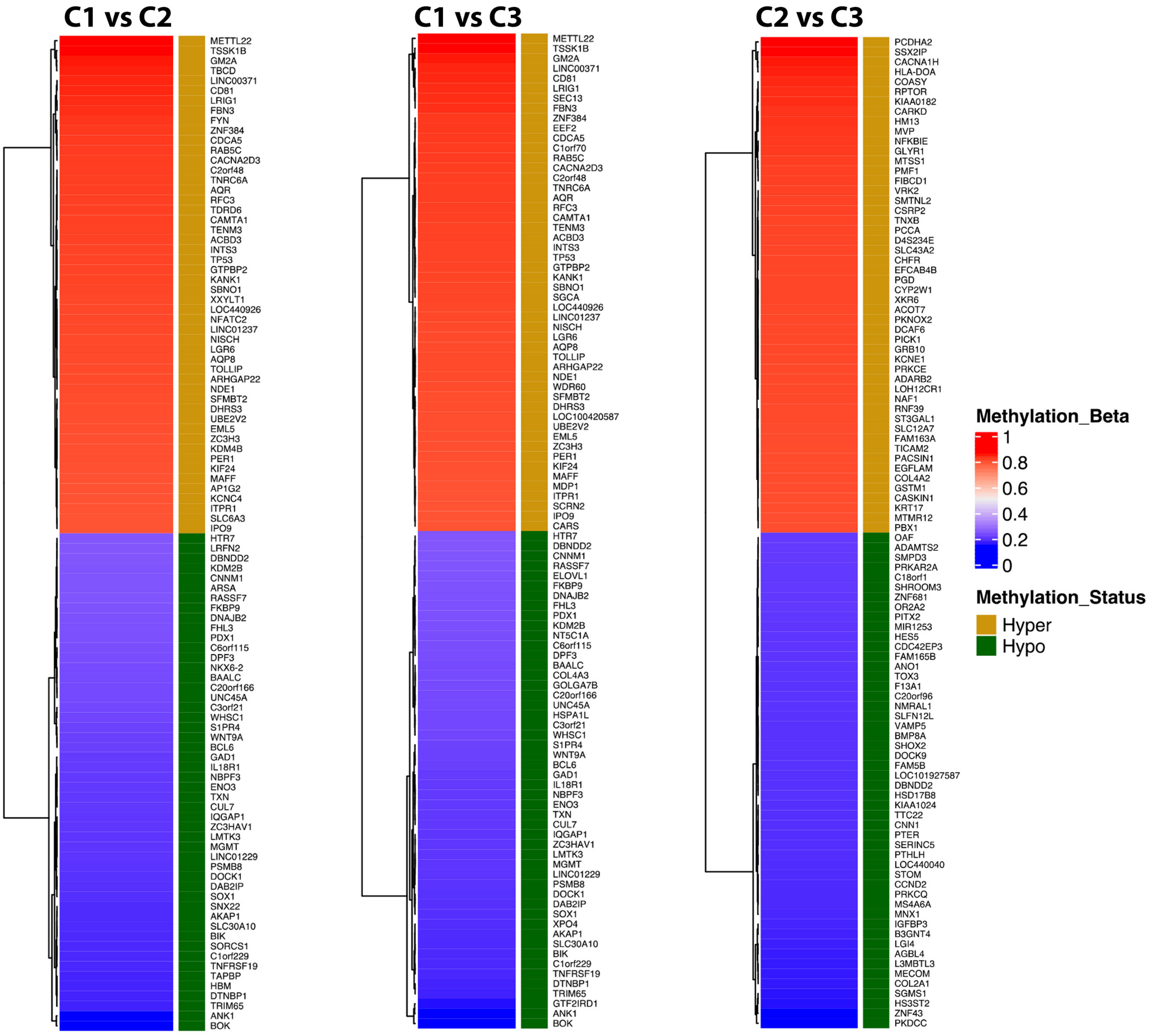
C1 vs C2
C1 vs C3
C2 vs C3
Methylation_Beta
1
0.8
0.6
0.4
0.2
0
Methylation_Status
Hyper
Hypo

Figure S4

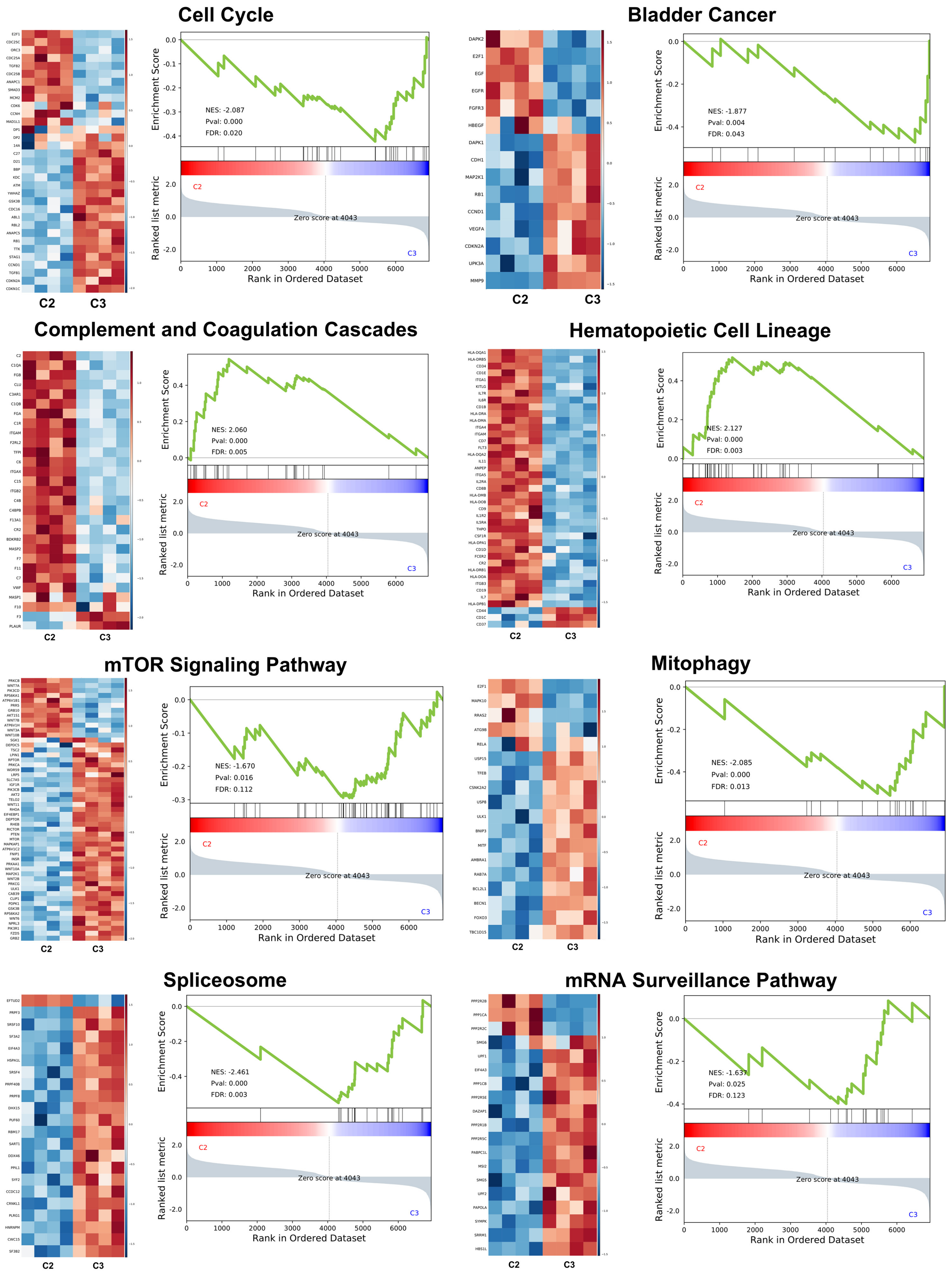

Cell Cycle
NES: -2.087
Pval: 0.000
FDR: 0.020
Bladder Cancer
NES: -1.877
Pval: 0.004
FDR: 0.043
Complement and Coagulation Cascades
NES: 2.060
Pval: 0.000
FDR: 0.005
Hematopoietic Cell Lineage
NES: 2.127
Pval: 0.000
FDR: 0.003
mTOR Signaling Pathway
NES: -1.670
Pval: 0.016
FDR: 0.112
Mitophagy
NES: -2.085
Pval: 0.000
FDR: 0.013
Spliceosome
NES: -2.461
Pval: 0.000
FDR: 0.003
mRNA Surveillance Pathway
NES: -1.637
Pval: 0.025
FDR: 0.123
Enrichment Score
Ranked list metric
Rank in Ordered Dataset
Zero score at 4043
C2
C3

Figure S5

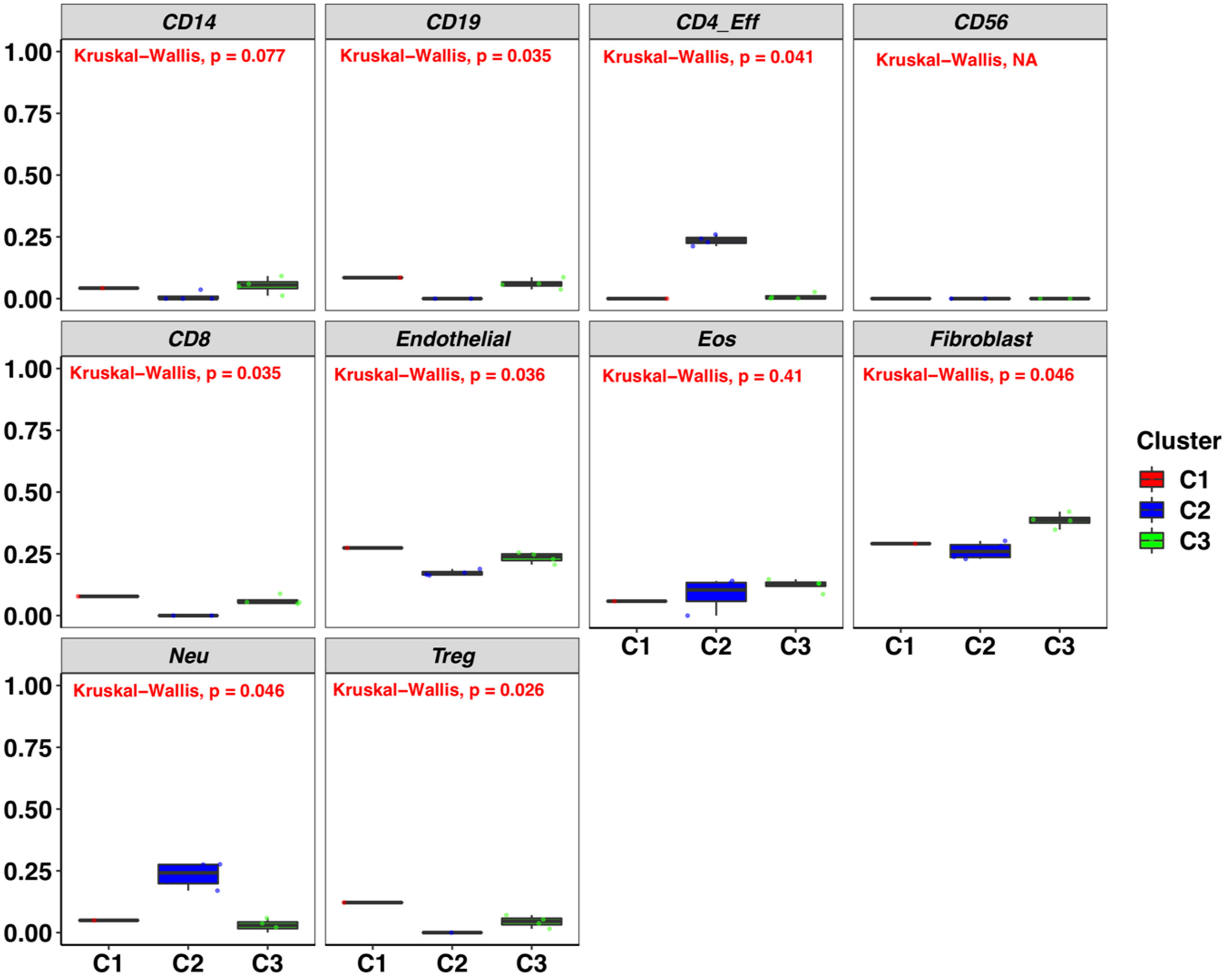
CD14
Kruskal–Wallis, p = 0.077
CD19
Kruskal–Wallis, p = 0.035
CD4_Eff
Kruskal–Wallis, p = 0.041
CD56
Kruskal–Wallis, NA
CD8
Kruskal–Wallis, p = 0.035
Endothelial
Kruskal–Wallis, p = 0.036
Eos
Kruskal–Wallis, p = 0.41
Fibroblast
Kruskal–Wallis, p = 0.046
Neu
Kruskal–Wallis, p = 0.046
Treg
Kruskal–Wallis, p = 0.026
1.00
0.75
0.50
0.25
0.00
C1
C2
C3
Cluster
C1
C2
C3

Supplemental Table S1

TOP HYPERMETHYLATED GENES DETERMINED BY PAIRWISE COMPARISONS BETWEEN CLUSTERS

	C1 vs C2 only	C1 vs C3 only	C2 vs C3 only	(C1 vs C2) and (C1 vs C3)	(C1 vs C2) and (C2 vs C3)	(C1 vs C3) and (C2 vs C3)	(C1 vs C2) and (C1 vs C3) and (C2 vs C3)
1	AP1G2	C1orf70	ACOT7	ACBD3	**None**	**None**	**None**
2	FYN	CARS	ADARB2	AQP8			
3	KCNC4	EEF2	CACNA1H	AQR			
4	KDM4B	LOC100420587	CARKD	ARHGAP22			
5	NFATC2	MDP1	CASKIN1	C2orf48			
6	SLC6A3	SCRN2	CHFR	CACNA2D3			
7	TBCD	SEC13	COASY	CAMTA1			
8	TDRD6	SGCA	COL4A2	CD81			
9	XXYLT1	WDR60	CSRP2	CDCA5			
10			CYP2W1	DHRS3			
11			D4S234E	EML5			
12			DCAF6	FBN3			
13			EFCAB4B	GM2A			
14			EGFLAM	GTPBP2			
15			FAM163A	INTS3			
16			FIBCD1	IPO9			
17			GLYR1	ITPR1			
18			GRB10	KANK1			
19			GSTM1	KIF24			
20			HLA-DOA	LGR6			
21			HM13	LINC00371			
22			KCNE1	LINC01237			
23			KIAA0182	LOC440926			
24			KRT17	LRIG1			
25			LOH12CR1	MAFF			
26			MTMR12	METTL22			
27			MTSS1	NDE1			
28			MVP	NISCH			
29			NAF1	PER1			
30			NFKBIE	RAB5C			
31			PACSIN1	RFC3			
32			PBX1	SBNO1			
33			PCCA	SFMBT2			
34			PCDHA2	TENM3			
35			PGD	TNRC6A			
36			PICK1	TOLLIP			
37			PKNOX2	TP53			
38			PMF1	TSSK1B			
39			PRKCE	UBE2V2			
40			RNF39	ZC3H3			
41			RPTOR	ZNF384			
42			SLC12A7				
43			SLC43A2				
44			SMTNL2				
45			SSX2IP				
46			ST3GAL1				
47			TICAM2				
48			TNXB				
49			VRK2				
50			XKR6				

TOP HYPOMETHYLATED GENES DETERMINED BY PAIRWISE COMPARISONS BETWEEN CLUSTERS

	C1 vs C2 only	C1 vs C3 only	C2 vs C3 only	(C1 vs C2) and (C1 vs C3)	(C1 vs C2) and (C2 vs C3)	(C1 vs C3) and (C2 vs C3)	(C1 vs C2) and (C1 vs C3) and (C2 vs C3)
1	ARSA	COL4A3	ADAMTS2	AKAP1	**None**	**None**	DBNDD2
2	HBM	ELOVL1	AGBL4	ANK1			
3	LRFN2	GOLGA7B	ANO1	BAALC			
4	NKX6-2	GTF2IRD1	B3GNT4	BCL6			
5	SNX22	HSPA1L	BMP8A	BIK			
6	SORCS1	NT5C1A	C18orf1	BOK			
7	TAPBP	XPO4	C20orf96	C1orf229			
8			CCND2	C20orf166			
9			CDC42EP3	C3orf21			
10			CNN1	C6orf115			
11			COL2A1	CNNM1			
12			DOCK9	CUL7			
13			F13A1	DAB2IP			
14			FAM165B	DNAJB2			
15			FAM5B	DOCK1			
16			HES5	DPF3			
17			HS3ST2	DTNBP1			
18			HSD17B8	ENO3			
19			IGFBP3	FHL3			
20			KIAA1024	FKBP9			
21			L3MBTL3	GAD1			
22			LGI4	HTR7			
23			LOC101927587	IL18R1			
24			LOC440040	IQGAP1			
25			MECOM	KDM2B			
26			MIR1253	LINC01229			
27			MNX1	LMTK3			
28			MS4A6A	MGMT			
29			NMRAL1	NBPF3			
30			OAF	PDX1			
31			OR2A2	PSMB8			
32			PITX2	RASSF7			
33			PKDCC	S1PR4			
34			PRKAR2A	SLC30A10			
35			PRKCQ	SOX1			
36			PTER	TNFRSF19			
37			PTHLH	TRIM65			
38			SERINC5	TXN			
39			SGMS1	UNC45A			
40			SHOX2	WHSC1			
41			SHROOM3	WNT9A			
42			SLFN12L	ZC3HAV1			
43			SMPD3				
44			STOM				
45			TOX3				
46			TTC22				
47			VAMP5				
48			ZNF43				
49			ZNF681				
50							
